# Supplementary material for: The association between chrononutrition behaviors and muscle health among older adults: The study of muscle, mobility and aging
Source: Aging Cell. 2023 Dec 7;23(6):e14059. doi: 10.1111/acel.14059 (PMC11166361; doi:10.1111/acel.14059)
Supplement: Supplementary file 1 — Figure S1. [file ACEL-23-e14059-s001.docx]

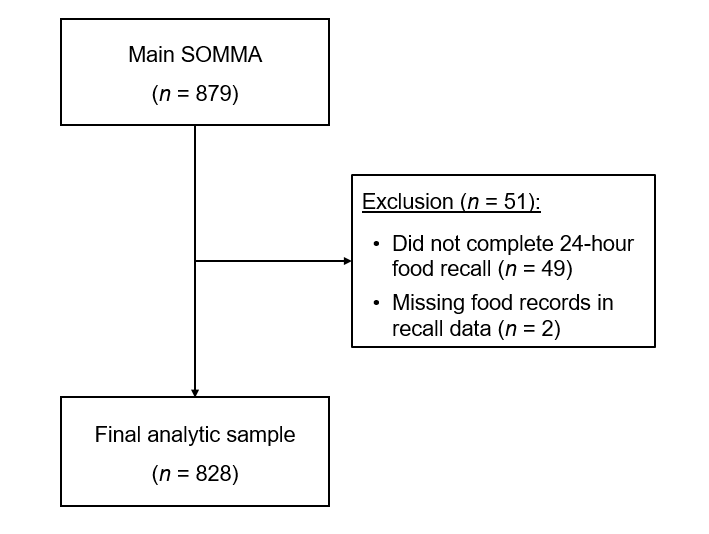


**eFigure 1** Flowchart depicting the exclusion and inclusion of SOMMA study participants in the analysis. SOMMA, Study of Muscle, Mobility, and Aging.
